# Cardiorespiratory phase synchronization maturational trajectory: biomarker of autonomic nervous system development in preterm infants

**DOI:** 10.1038/s41390-026-04783-1

**Published:** 2026-02-05

**Authors:** Narayanan Krishnamurthi, Casey M. Rand, Raye-Ann deRegnier, Erin K. Lonergan, Michael S. Carroll, Lynn Boswell, Ryne Estabrook, Susan M. Slattery, Aaron Hamvas, Debra E. Weese-Mayer

**Affiliations:** 1 Center for Autonomic Medicine in Pediatrics (CAMP), Department of Pediatrics, Ann & Robert H Lurie Children’s Hospital of Chicago and Stanley Manne Children’s Research Institute, Chicago, IL, USA.; 2 Division of Neonatology, Department of Pediatrics, Ann & Robert H Lurie Children’s Hospital of Chicago and Stanley Manne Children’s Research Institute, Chicago, IL, USA.; 3 Department of Pediatrics, Northwestern University Feinberg School of Medicine, Chicago, IL, USA.; 4 Department of Psychology, University of Illinois at Chicago, Chicago, IL, USA.; 5 Beatrice Cummings Mayer Endowed Professor of Pediatric Autonomic Medicine, Northwestern University Feinberg School of Medicine, Chicago, IL, USA.; 6 These authors contributed equally: Aaron Hamvas, Debra E. Weese-Mayer.

## Abstract

**BACKGROUND:**

Cardiorespiratory coupling (cardiovascular-respiratory interactions) is a marker of autonomic nervous system function and maturation. We hypothesize that differences in autonomic maturation are associated with disparate outcomes of premature infants.

**METHODS:**

We collected continuous bedside cardiorespiratory data from birth to 40 weeks post-menstrual age (PMA) for 191 preterm infants born between 23–0/7 weeks to 28–6/7 weeks of gestation. Using the respiratory and ECG wave forms, we calculated cardiorespiratory phase synchronization (CRPS) as a measure of cardiorespiratory coupling. Using linear mixed effects modeling, we studied the trajectory of CRPS as a function of PMA and chronological age (CA) and any difference between groups separated by discharge status, respiratory outcome and neurological outcomes at the 40th week PMA, and different gestational ages.

**RESULTS:**

CRPS showed a decline reaching a nadir at approximately 3 weeks of age followed by a gradual increase toward term. The infants born at later GA showed higher values and steeper increases than infants of lower GA. Contrary to our hypothesis, the trajectory of the increase was similar regardless of outcome assessed.

**CONCLUSION:**

Autonomic maturation following preterm birth shows a fixed predictable pattern that appears to be independent of respiratory or neurologic outcomes or clinicians’ assessment of readiness for discharge.

## INTRODUCTION

Cardiorespiratory coupling is the interaction of the cardiovascular and respiratory systems to maintain physiologic homeostasis. In the transition to postnatal life, infants must initiate regular respirations and coordinate these with postnatal circulatory changes to maintain physiologic homeostasis. As the autonomic nervous system (ANS) is in a rapid state of development in the third trimester, cardiorespiratory coupling may be adversely impacted in infants born preterm. The unmyelinated parasympathetic system develops first in the fetus, followed by emergence of the sympathetic system. Sympathetic activity predominates early and increases throughout fetal growth, whereas parasympathetic activity increases most rapidly in the third trimester and continues to mature for months after birth.^1–3^ Preterm birth interrupts this sequence of events, resulting in an immature ANS. The underdeveloped respiratory control centers and immature chemoreceptor responses contribute to respiratory instability, manifesting as apnea, bradycardia, and intermittent hypoxemia.^4,5^ The degree and nature of cardiorespiratory coupling serve as a direct reflection of an infant’s neurodevelopmental maturity and maturation of the ANS.^6^ Moreover, it is thought to improve the efficiency of pulmonary gas exchange,^7^ reduce cardiac work,^5^ stabilize systemic blood flow/arterial blood pressure, and optimize oxygen delivery.^8^

Cardiorespiratory coupling has been studied using traditional time and frequency domain analyses,^9,10^ information theory-based and model-based methods^11–13^ and many other nonlinear techniques.^14^ However, quantifying cardiorespiratory coupling using cardiorespiratory phase synchronization (CRPS) has many advantages, particularly in hospitalized premature infants, including the ability to detect weak coupling, insensitivity to noise and missing data, insight into temporal dynamics, distinction from respiratory sinus arrhythmia, and the ability to detect nonlinear interaction.^11,15,16^

CRPS has been widely used and specifically applied to study physiology in premature and newborn infants,^6,10,17–19^ during different sleep states,^7,20^ sleep apnea,^21–24^ myocardial infarction,^25,26^ heart transplantation,^27^ hypoxemia,^28^ etc. Cardiorespiratory coupling measured as CRPS is weak or nearly absent at the earliest gestational ages (26–31 weeks GA) and increases with postnatal development) due to maturation of respiratory control.^6,17,29–31^ Delayed parasympathetic maturation and lower heart rate variability might lead to a lack of CRPS in very preterm babies.^5,11^ Taken together, CRPS provides a quantitative look into the functional maturation of the ANS in preterm infants. The strength, directionality, and patterns of this coupling evolve with age and are influenced by sleep states and clinical conditions, reflecting the ongoing development of crucial brainstem networks that are essential for stable cardiorespiratory function. Although Clark, et al. ^6^ found an increase in cardiorespiratory coupling over time in preterm infants as early as 26 weeks of gestation, this developmental trajectory has not been reported for infants born as early as 23 weeks of gestation, nor investigated in relationship to pulmonary outcomes, early brain injury, and other prematurity-associated outcomes.

The NHLBI-funded Prematurity-related Ventilatory Control (PreVent) study (ClinicalTrials.gov identifier NCT03174301) included continuous recording of the electrocardiogram, respiratory impedance, and oxygen saturations from the bedside cardiorespiratory monitors from 717 infants born before 29 weeks gestation across 5 level IV NICUs.^32^ This unique study design empowered unprecedented characterization of physiologic trajectories of apnea, periodic breathing, intermittent hypoxemia, and bradycardia by gestational age (GA) and with advancing chronological age.^33^ Advanced analytics additionally allowed for identification of physiologic biomarkers both to predict adverse pulmonary outcomes and death in this cohort and to examine metrics reflective of cardiorespiratory maturation.^4^ Assessing longitudinal CRPS in this cohort offers an opportunity to advance our understanding of trajectories of cardiorespiratory autonomic maturation in infants born extremely preterm and may offer a biomarker of early outcomes in the NICU.

Using the extensive physiologic data available through a single-center cohort of the Pre-Vent study, we therefore hypothesized that the absolute CRPS and the trajectory of maturation (as measured by the rate of CRPS) increase over time, would be reduced in infants born extremely preterm with unfavorable pulmonary outcomes or severe brain injury. We further hypothesized that infants discharged at younger PMA, considered a reflection of clinicians’ assessments of physiologic maturity and readiness for discharge, might show a steeper trajectory for CRPS maturation.

## METHODS

### Study population

Participants were recruited from NICUs at Prentice Women’s Hospital and the adjacent Ann & Robert H. Lurie Children’s Hospital (LCH) of Chicago, together the LCH-Northwestern University single site in the Pre-Vent study. Participants were born between 23–0/7 weeks to 28–6/7 weeks of gestation and were less than 1 week of age at study entry (Fig. 1). Infants with congenital or chromosomal anomalies and infants who were considered unlikely to survive by the clinical team were excluded. Institutional Review Board approval was obtained with waiver of consent. Oversight was provided by an observational safety monitoring board appointed by NHLBI.

### Data collection

Electrocardiogram (ECG) and chest transthoracic impedance data (TTI) were collected continuously from NICU admission (≤ 7 days of birth) until death or discharge, up to 40 weeks post menstrual age (PMA). The ECG data were collected at 250 Hz and TTI at 62.5 Hz using NICU bedside Philips monitor (Andover, MA) and either MediCollector (Boston, MA) or BedMaster (Anandic Medical Systems, Feuerthalen, Switzerland) data collection systems.

### Data processing

The 24-hour/day continuous ECG was the source for heart rate data and chest TTI signals were the source for respiratory excursion data to collectively investigate CRPS. To avoid noisy TTI data,^6,34^ TTI data segments were utilized only if the following criteria were met (Fig. 2): (1) the data segment was not a flat line; (2) the data saturation (flat line) was not more than 10% for a given 30-sec data segment; and (3) the data did not contain any apneic episodes (≥ 20 sec).^4^ Acceptable TTI data segments were low-pass filtered using zero-phase (not altering the phase information) with a cutoff at 4 Hz. The peaks of the TTI data were obtained using the Automatic Multiscale Peak Detection algorithm,^35^ which is non-parametric and robust for noisy, periodic, and quasi-periodic data. The peak timings of the TTI data were used to calculate inter-breath intervals for a given 30-sec segment and ensured that at least 18 seconds of the 30-sec segment consisted of valid breaths. A given segment was considered valid if the normalized standard deviation of the inter-breath intervals was less than 0.25, and > 85% of the intervals were within 0.5 to 1.5 times the median. Finally, for segments with a respiratory rate greater than 0.25 Hz, a template breath was created and compared against the individual breaths. Segments were retained for CRPS calculations only if the mean correlation between the template and the individual breaths of the segment exceeded 0.75, ensuring that breaths within the segment exhibited similar morphology.^34^

Good quality 30-sec TTI data segments were used to assess the corresponding ECG segment.^36^ R-wave peaks were detected using the “jqrs” algorithm part of the open-source MATLAB package.^37^ The phases of the respiratory signal (using Hilbert Transform) corresponding to the R-peak timings were used to calculate phase synchronization. For a given m number of breaths and n number of R peaks, the respiratory signal phases $\phi_{i}$ at which R peaks of ECG occurred were collected. If the R peaks happen at a consistent phase of the respiration, $\phi_{i}$ will cluster which means strong coupling. If the R peaks occur randomly throughout the m respiratory cycles, then $\phi_{i}$ will be uniformly spread meaning no coupling. The $\phi_{i}^{'}s$ are mapped to a unit circle resulting in a unit vector pointing in the direction of $\phi_{i}$. Then, the magnitude of the average of these vectors is obtained (synchronization index, SI) using the first Fourier mode.^19^ If the $\phi_{i}^{'}s$ are always aligned, all the vectors will point in the same direction resulting a maximum synchronization value of 1 in a noise-free case and if the phases are random, then the vectors will cancel out resulting in a synchronization value of 0. For a given 30-sec data segment, the phase synchronization was calculated for different n:m ratios (n number of R-peaks within m breaths), and the largest synchronization value among different n:m ratios was selected.^24^

Surrogate data analysis was performed to ensure that the observed largest phase synchronization for a particular 30-second data segment was not just due to a random origin. For each 30-second segment, we calculated the phase synchronization value for 100 different realizations of phase-randomized surrogate data (where the phase information of respiratory data was randomized) but preserving the distribution and power spectral density of the respiratory data. For each realization of surrogate data, we calculated the phase synchronization value for various n:m ratios (m=1 *to 5*, n=m+1 *to 35*). The largest synchronization index of the original data was considered significant only if that value was at least >95th percentile of the mean synchronization index obtained from the surrogates. Only significant CRPS values (obtained for 30-second segments) were used for investigating trends among participants across PMA (until the 40th week) and CA for any group comparisons.

### Statistical analysis

CRPS values were evaluated with advancing postnatal age [chronological age (CA)] and PMA and assessed for potential differences between infants based on GA (grouped as 23–24, 25–26, and 27–28 weeks), age of discharge (before or after 40 weeks PMA), severe brain injury, and favorable vs. unfavorable pulmonary outcomes. As in previous Pre-Vent studies, severe brain injury was defined as grade 3–4 intraventricular hemorrhage, post-hemorrhagic hydrocephalus, and cystic periventricular leukomalacia. An unfavorable pulmonary outcome was defined as a requirement for respiratory support or respiratory specific medications at 40 weeks PMA or discharged before 40 weeks on respiratory specific medications or other respiratory support.^4,32^ For each PMA/CA day, the median of CRPS values across available participants (for that particular day) was calculated and used for linear mixed-effect models with random intercept. The trajectories and 95% confidence level shown in Figs. 3–6 were obtained using a non-parametric regression method, locally estimated scatterplot smoothing (loess). All analyses were conducted using linear mixed effects models using the *lme4* package.^38^ All initial models included linear and quadratic effects of one measure of age, either PMA or CA. These models were then updated to include (a) a main effect of one of our four grouping variables, (b) an interaction between group and the linear age trend, and (c) an interaction between group and the quadratic or non-linear age trend. Comparisons between models were made via likelihood ratio test, which provides a $\chi^{2}$ *p*-value.

## RESULTS

### Study population

191 extremely preterm-born infants participated in this single-site cohort. Clinical and demographic characteristics are shown in Table 1. Of these 191 participants, 137 contributed data to the multi-site Pre-Vent dataset^4,33^ with dates of inclusion May 2018 through December 2020, and an additional 54 infants were sequentially recruited locally under the same protocol with dates of inclusion January 2021 through December 2021. Altogether, 67 infants (35%) were less than 26 weeks GA at birth. Characteristics of these two groups are compared in Supplemental Table S1. Some clinical characteristics differed between the earlier and later groups, but their impact on our results, if any, cannot be determined.

### Data suitability for CRPS analysis

Data from 175 participants were available (Fig. 1). The median number of days of data available per subject was 63.4 (range: 5–205). A total ~11,100 days of non-overlapping 30-sec data segments were available, of which ~2400 days of respiratory TTI data were identified as sufficient quality to be considered for CRPS analysis. A median of five hundred 30-second good-quality data segments (~ 4 hours) per day were available to calculate CRPS. High-quality segments generally increased with maturation and decreased during periods coinciding with routine hands-on care and feeding times in our NICU (data not shown). Of these good quality segments, about 12% of the data segments’ (~ 2400 days) CRPS values were identified as significant based on the surrogate data analysis. The number of segments that showed significant CRPS (as a percentage of the number of high-quality segments) decreased from 24 to 29 weeks PMA and 1 to 5 weeks CA and then increased gradually (data not shown). The *2:1* ratio was found to be the most frequently occurring n:m pair across subjects and PMA/CA days.

### Changes in CRPS by chronological, gestational, and postmenstrual age

Evaluation of CRPS by PMA (Fig. 3a) and CA (Fig. 3b) shows an initial decline during the first postnatal weeks that was followed by a linear increase over time for the remainder of the recording period (until NICU discharge).

### Gestational age group

When CRPS was evaluated across PMA for each GA group (23–24, 25–26, and 27–28 weeks GA; Fig. 4a) there were significant linear ($\chi_{2}^{2}=9.129$, *p* = 0.010) and quadratic interactions (PMA : $\chi_{2}^{2}=35.772$, *p* < 0.001) of GA group and age, with main effect of GA ($\chi_{2}^{2}=0.886$, *p* = 0.642). These trends are difficult to interpret due to the confounding of the GA grouping variable and data availability for the PMA variable. For CRPS evaluated across CA (Fig. 4b), there is both a main effect of GA group ($\chi_{2}^{2}=7.29$, *p* = 0.027) and a linear interaction ($\chi_{2}^{2}=59.311$, *p* < 0.001), but no quadratic age by group interaction, CA : $\chi_{2}^{2}=3.982$, *p* = 0.137). CA trends show comparable CRPS decreases over the first 2–4 weeks of life in all GA groups, with significantly stronger increases (*p* < 0.001) in CRPS for later GAs. Whenever there is a significant interaction between two fixed factors (PMA/CA and GA groups in this case), it indicates that the slopes of any two GA group trajectories are significantly different from each other.

### Discharge status

Longitudinal CRPS trends by discharge age (> 40 weeks vs ≤40 weeks) by PMA and CA are shown in Fig. 5a and b, respectively. There is a main effect of discharge group for the PMA model ($\chi_{1}^{2}=5.765$, *p* = 0.016), but not for the CA model ($\chi_{1}^{2}=3.026$, *p* = 0.082). There are no significant interactions for either linear (PMA : $\chi_{1}^{2}=2.270$, *p* = 0.132; CA : $\chi_{1}^{2}=1.506$, *p* = 0.212) or quadratic terms (PMA : $\chi_{1}^{2}=3.028$, *p* = 0.082; CA : $\chi_{1}^{2}=2.900$, *p* = 0.089). Discharge >40 weeks shows significantly higher CRPS than discharge ≤40 weeks for the PMA age model.

### Intracranial imaging abnormalities

There were no main effects between CRPS by PMA or CA and the presence of significant intracranial hemorrhage or periventricular leukomalacia with imaging (data not shown).

### Favorable vs. unfavorable respiratory outcome

Longitudinal CRPS trends for infants with favorable vs. unfavorable outcome by PMA and CA are shown in Fig. 6a, b, respectively. Results were the same regardless of whether PMA or CA was used as a measure of time. There was a significant main effect of favorable vs. unfavorable outcome group (PMA : $\chi_{1}^{2}=8.213$, *p* = 0.004; CA : $\chi_{1}^{2}=4.365$, *p* = 0.037), as well as significant interactions between outcome group and the quadratic trend, indicating group differences in the non-linear trend (PMA: $\chi_{1}^{2}=6.882$, *p* = 0.009; CA : $\chi_{1}^{2}=4.160$, *p* = 0.041). There were no differences in the outcome group by linear age trend terms (PMA : $\chi_{1}^{2}=0.854$, *p* = 0.355; CA : $\chi_{1}^{2}=3.236$, *p* = 0.072). These effects show significantly lower CRPS trends in the favorable outcomes group earlier, which disappears with increasing PMA and CA.

## DISCUSSION

The maturation of the parasympathetic and sympathetic divisions of the ANS follows distinct developmental trajectories. The unmyelinated parasympathetic system develops first, followed by the emergence of the sympathetic system. In the early fetal growth, sympathetic activity predominates, whereas in the third trimester and after birth, parasympathetic activity increases most rapidly.^1–3^ During the late fetal and early postnatal period, complex connections between supratentorial and brainstem ANS centers and the hypothalamic-pituitary-adrenal (HPA) axis also emerge, enabling the regulation of respiratory, cardiovascular, and gastrointestinal functions in response to environmental exposures and stressors.^30,39^ Although the Pre-Vent study did not enroll term infants for direct comparison, previous studies suggest that by term-equivalent age, autonomic maturation is similar for those born extremely preterm and at term.^6,39^

We hypothesized that CRPS, as a measure of cardiorespiratory coupling, would be reduced in earlier GA infants, would increase over time, but would be diminished in preterm infants who experienced adverse neurologic or respiratory sequelae. This maturational pattern was observed with a decline in CRPS in the first weeks after birth, followed by a gradual increase over the duration of the neonatal hospitalization. Contrary to our hypothesis, the trajectory of the increase was similar regardless of the outcome assessed.

This observed decrease in CRPS in the first weeks after birth was unexpected but appears to be determined by chronological age rather than gestational age at birth (Fig. 4). The cardiovascular transition at birth is modulated by corticosteroids and catecholamines with a catecholamine surge occurring after umbilical cord clamping in both preterm and term infants.^40–43^ However, adrenal responsiveness is developmentally regulated and initially blunted until the number of beta-adrenergic cardiac and pulmonary receptors increases.^40^ We speculate that the early postnatal decline in CRPS reflects this neuroendocrine interplay, which subsequently stabilizes after birth.

There are few studies that evaluate the maturation of CRPS in infants born preterm. Our overall findings are similar to those of Clark et al. ^6^ who studied nearly two decades of NICU patient data and similarly showed that cardiorespiratory interactions increased with postnatal age. They found that the rate of increase was independent of GA, indicating that development of cardiorespiratory autonomic control is a highly conserved postnatal age-dependent phenomenon. However, the data in the Clark et al. ^6^ paper were collected over a decade ago and reflect the maturation patterns of a cohort of more stable and mature infants.

Although we expected that infants with unfavorable pulmonary outcomes or severe brain injury might have dysmaturational patterns compared with healthier preterm-born infants, this was not observed. Furthermore, we postulated that infants who were discharged at a younger PMA (a reflection of clinicians’ assessments of physiologic maturity and readiness for discharge) might show a steeper trajectory for CRPS maturation, but this also was not observed. These findings suggest that the maturation of autonomic function is preserved during early development even in the presence of intracranial hemorrhage or periventricular leukomalacia, perhaps due to its vital importance in the transition from fetal to extrauterine life.

It is unclear if differences in the magnitude of the CRPS values seen at the nadir and end of the study period in some of the groups have physiological implications, as there are no clear precedents for these numbers. Furthermore, infants born at 27–28 weeks were discharged earlier, on average, compared with those born at earlier GA, and the data proximal to discharge or 40 weeks may have been skewed by this. Additionally, there are no studies of CRPS in term infants that establish a baseline at 38–40 weeks PMA for comparison with our results.

Chest impedance data are inherently noisy and can be disrupted by movement or other environmental disturbances. We found that 21% of our data were of sufficient quality for analysis. For reference, Clark et al. ^6^ reported 33% of their NICU respiratory data were of good quality, however they used different methodology to verify data quality. Despite variations in signal quality, the volume of data available still provides insight into these measures of autonomic maturation.

Sleep state can be an important modifier of cardiorespiratory coupling. A limitation of the study is that we did not have the ability to capture the electroencephalographic data necessary to clearly define the sleep state. Additional limitations include the undefined role of respiratory support on CRPS and, likewise, the use of medications such as inotropic agents, corticosteroids, or caffeine. While we did not find differences in CRPS based on a cross-sectional analysis of invasively versus non-invasively ventilated infants (data not shown), infants were frequently alternating between modes and degree of support, and therefore, it is difficult to obtain sufficient longitudinal data to have more detailed insight. Finally, the “favorable” or “unfavorable” outcomes as outlined by the Pre-Vent consortium are respiratory-focused and may not be the most representative outcomes from which to identify differences in autonomic maturation. Cardiorespiratory and neurodevelopmental outcomes at later ages will be crucial to determine whether the subtle differences observed in early life autonomic maturational trajectories impact long-term outcomes. Studies are currently underway, specifically examining Pre-Vent infants at 5–7 years of age in the ongoing Post-Vent study.

In summary, this study represents the most longitudinal and comprehensive report to date in understanding autonomic maturation utilizing CRPS in premature infants. The complex algorithm applied to continuous data extracted from bedside monitors throughout postnatal growth to NICU discharge identified a chronologic age-associated and gestational age-independent change in CRPS that decreased in the first week and then gradually increased toward term gestation. These data suggest that the postnatal environment is critical for shaping sympathetic adaptation to extrauterine life. Continuous measures of cardiorespiratory interaction can measure developmental maturity in neonatal intensive care patients and help in decisions about developing illness and hospital discharge. Decreasing CRPS may precede clinically evident deterioration and may serve as an early detection tool. Irregular cardiorespiratory coupling is implicated in pathologies like apnea of prematurity and potentially Sudden Infant Death Syndrome, making phase coupling methods useful for early identification of possible risk states and early markers of cardiorespiratory dysregulation.

## Supplementary Material

Supplementary Table

**Supplementary information** The online version contains supplementary material available at https://doi.org/10.1038/s41390-026-04783-1.

## Figures and Tables

**Fig. 1:**
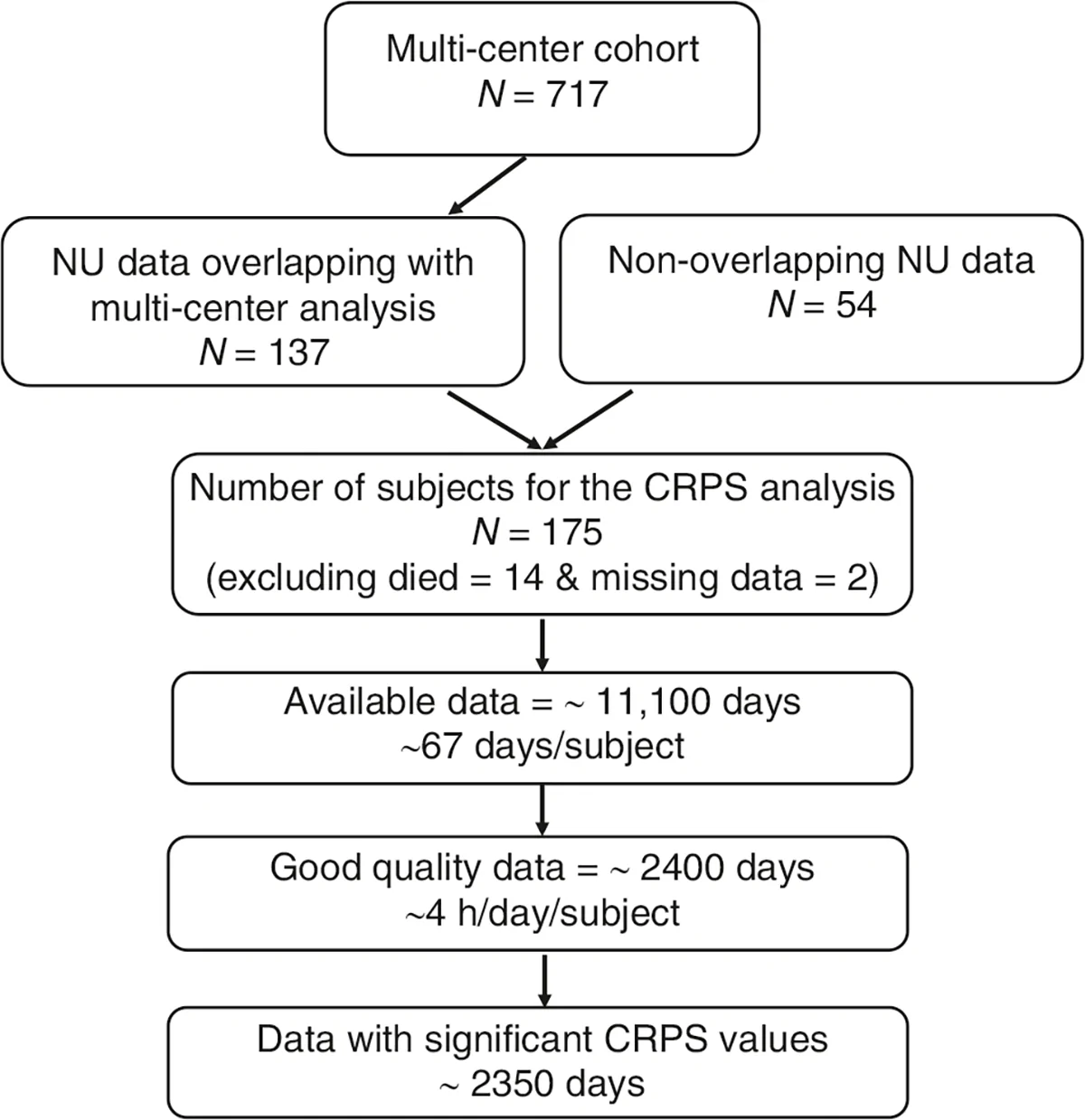
Cohort Availability and Data Processing Pipeline. Flow of participants through the study (overlapping and non-overlapping with the multi-center Pre-Vent cohort, and excluding the participants who died) with information on the availability of good quality data for the cardiorespiratory coupling analysis.

**Fig. 2:**
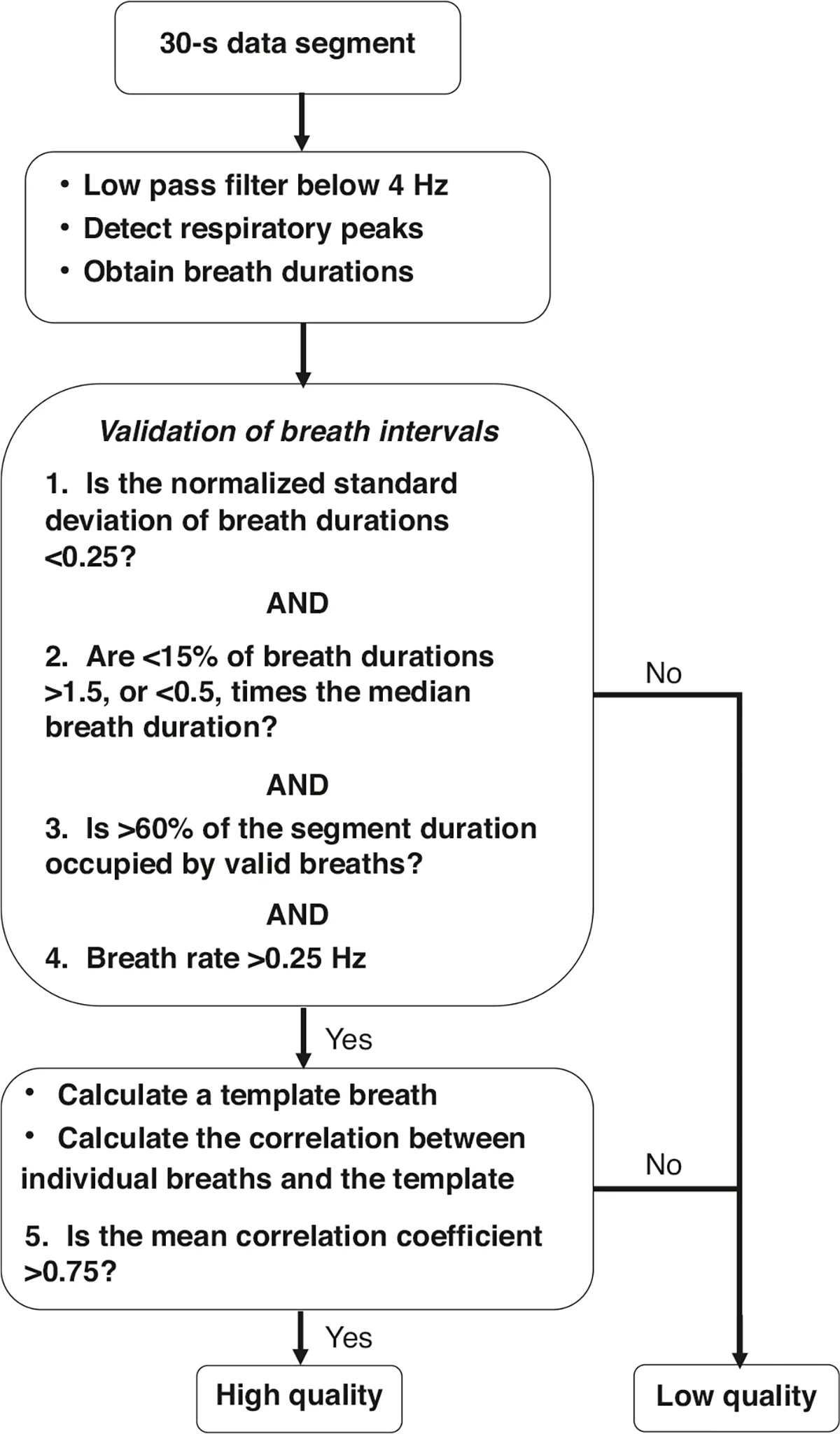
Schematic of Steps Involved in Identifying Good Quality Chest Transthoracic Impedance (TTI). TTI data was divided into (1) 30-sec segments, (2) filtered, (3) inter-breath-intervals were obtained and validated, and (4) each breath was matched with a template breath, to identify the quality of 30-sec TTI data segments. Only good quality TTI segments were used for the calculation of cardiorespiratory phase synchronization values.

**Fig. 3:**
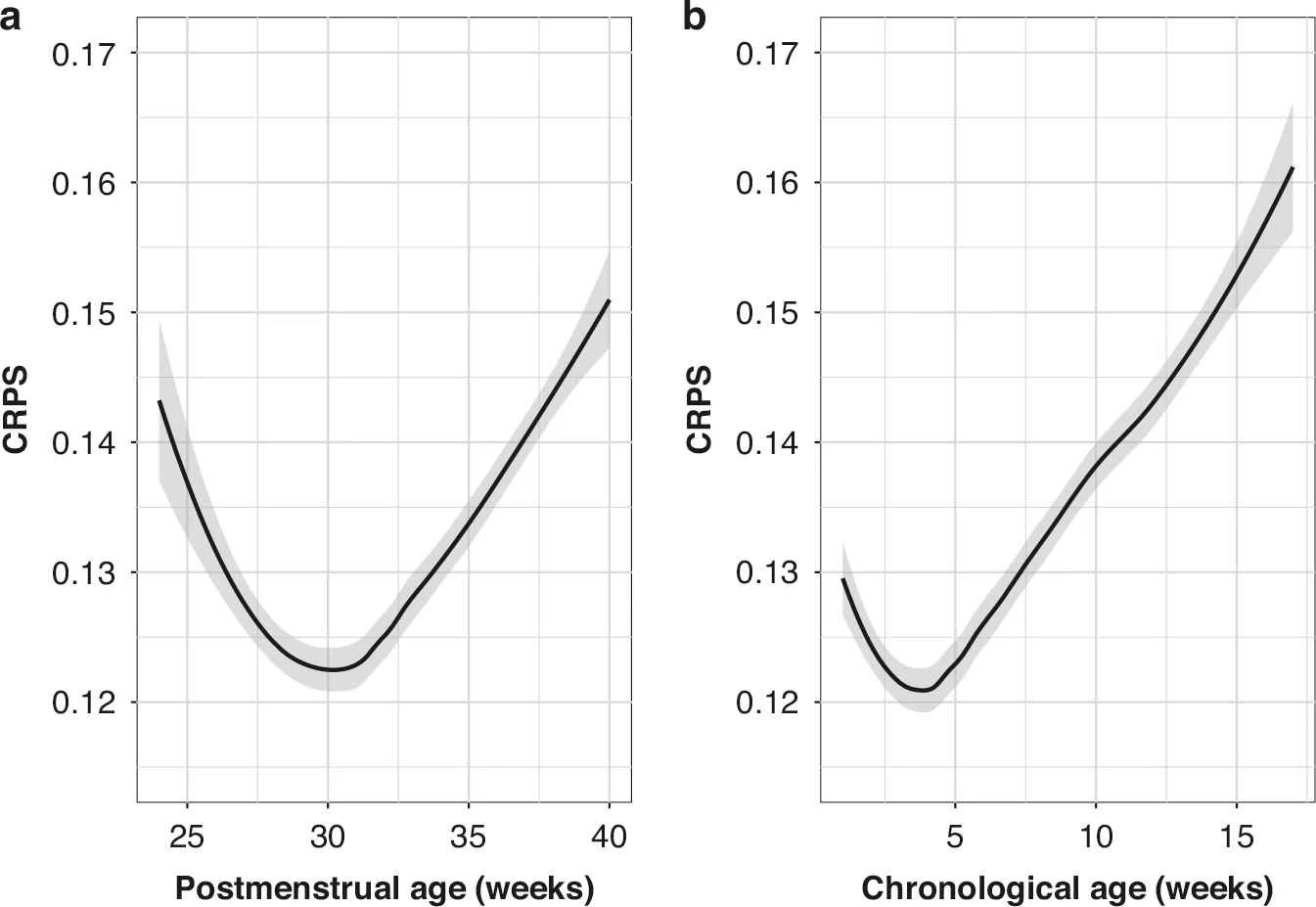
CRPS Trajectories for the Entire Cohort. Loess-smoothed trends in CRPS by postmenstrual age (**a**) and chronological age (**b**), *n* = 175. The bands about the CRPS trajectories represent 95% confidence intervals. loess: locally weighted regression smoothing.

**Fig. 4:**
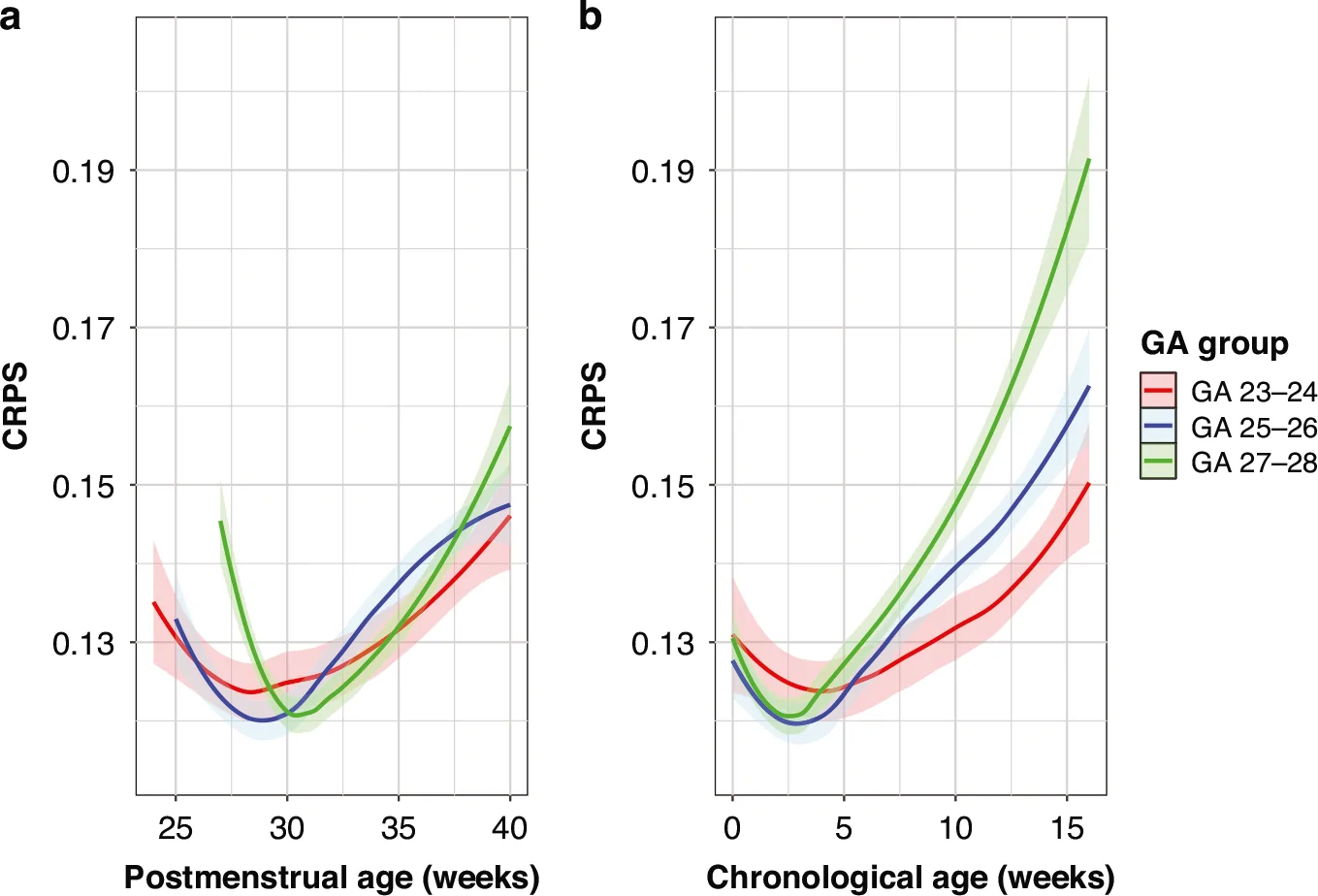
CRPS Trajectories Across Gestational Age Groups. Loess-smoothed trends in CRPS by postmenstrual age (**a**) and chronological age (**b**) as a function of gestational age groups (23–24 weeks, *n* = 24; 25–26 weeks, *n* = 54; 27–28 weeks, *n* = 97). Accompanying linear mixed models show a significant main effect of gestational group as well as interactions with the postmenstrual and chronological age trends. Trends are difficult to interpret for the postmenstrual age due to confounding between gestational age group and range of the gestational age variable. Chronological age trends show comparable decreases over the first four weeks of life, and significantly stronger increases in CRPS growth for later gestational ages.

**Fig. 5:**
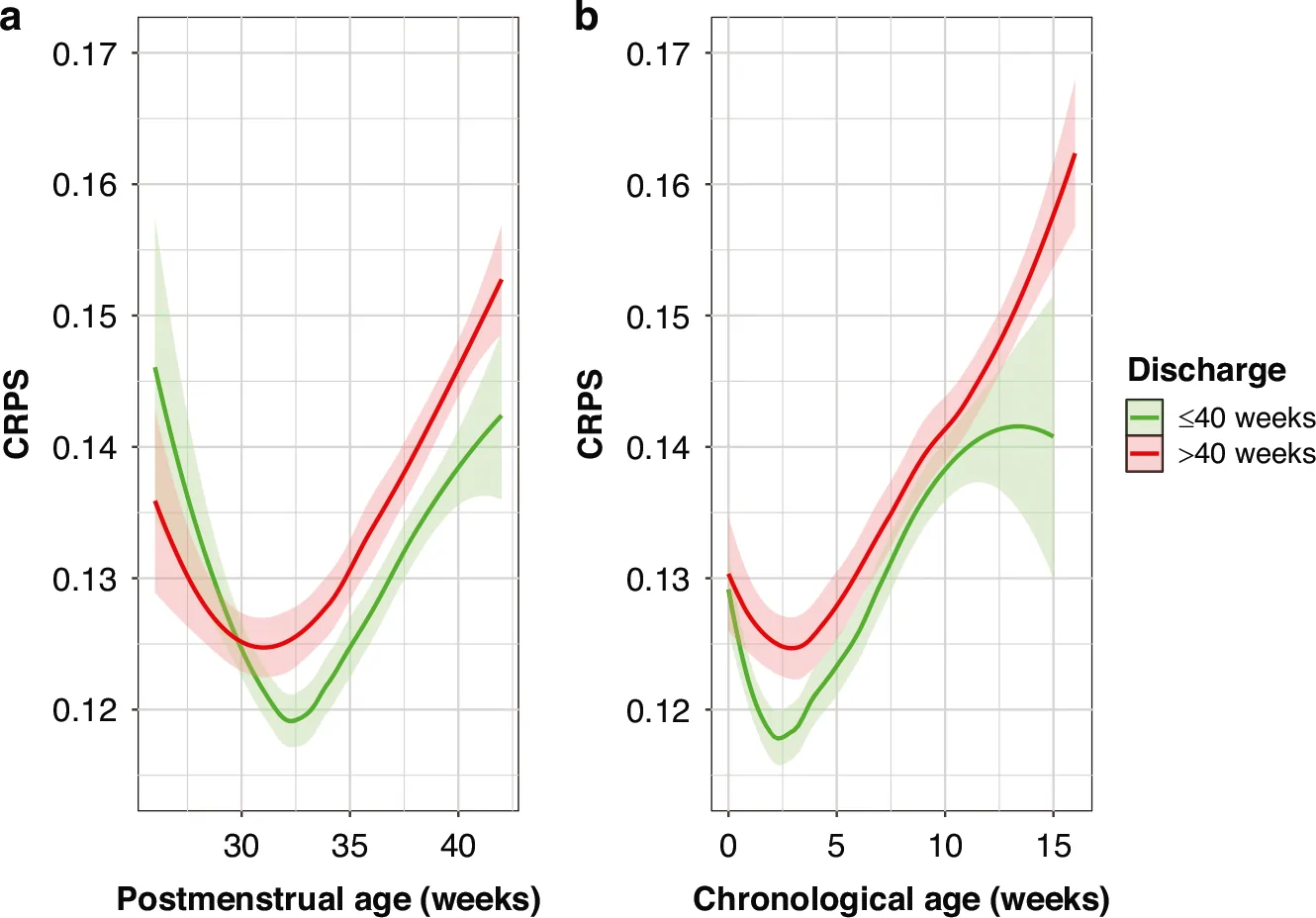
CRPS Trajectories By Discharge Status. Loess-smoothed trends in CRPS by postmenstrual age (**a**) and chronological age (**b**) as a function of discharge status groups (40 weeks or below, *n* = 91; Over 40 weeks, *n* = 84). Accompanying linear mixed models show a significant main effect of discharge status for postmenstrual age, but no effect for chronological age and no significant interactions between discharge status and trends for either age variable. Discharge after 40 weeks shows significantly higher CRPS than earlier discharge for the postmenstrual age model.

**Fig. 6:**
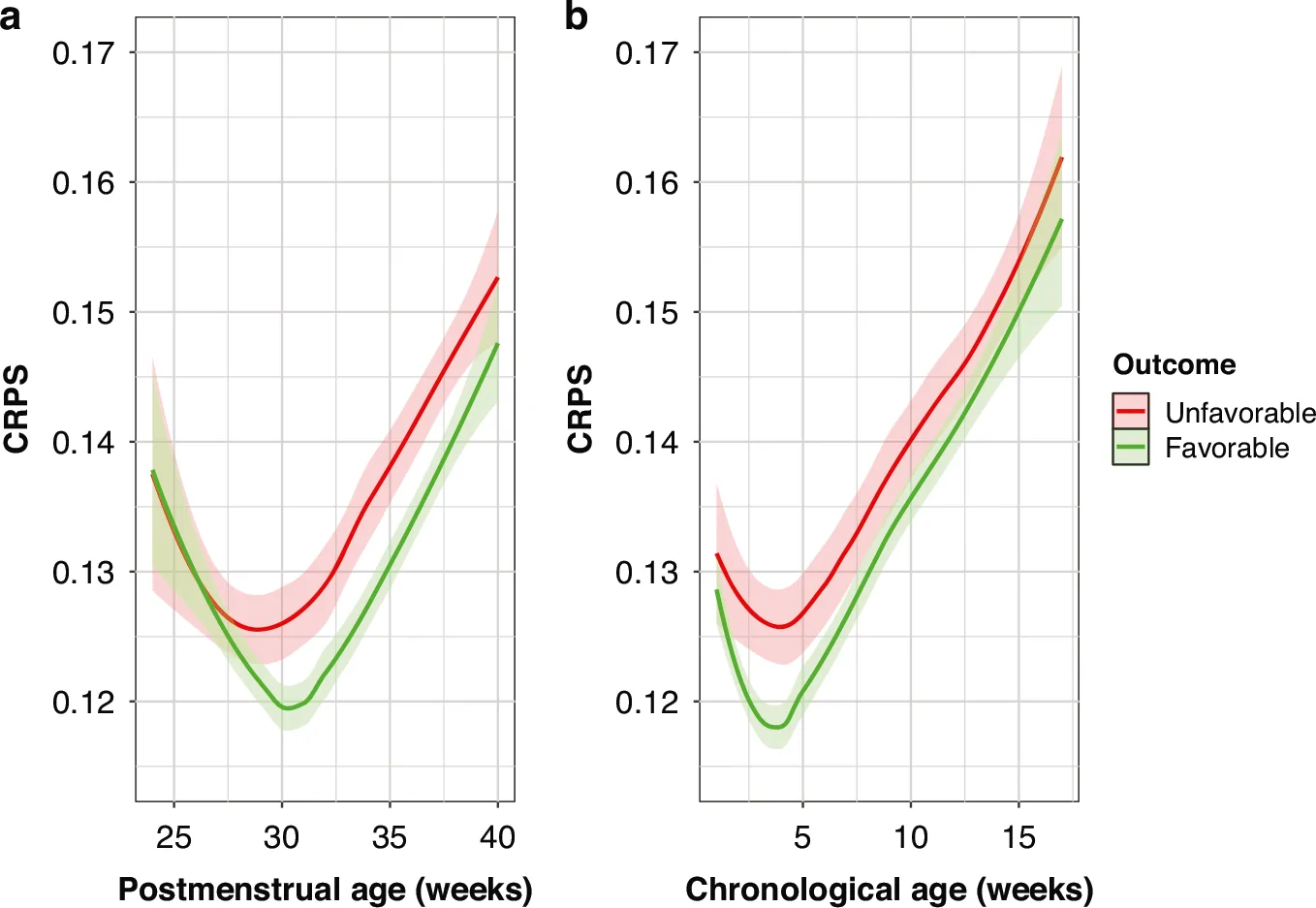
CRPS Trajectories Stratified by Outcome. Loess-smoothed trends in CRPS by postmenstrual age (**a**) and chronological age (**b**) as a function of Favorable (*n* = 107) vs Unfavorable (*n* = 68) outcome groups. Accompanying linear mixed models show a significant main effect of outcome group as well as interactions with the postmenstrual and chronological age trends. These effects show significantly lower CRPS in the Favorable outcomes group earlier, which disappears with increasing post menstrual age and chronological age.

**Table 1.** Characteristics of the Full Site Cohort.

Cohort Characteristics, *N* = 191	*N*
Birth weight (g), mean (SD)	970 (295)
<10th percentile weight (Fenton)	17
Gestational age (weeks), mean (SD)	26.3 (1.55)
< 26 weeks at birth	67 (35%)
Sex (female)	85 (46%)
Multiple gestation	57 (29.8%)
Born outside study center	30 (15.7%)
Clinical chorioamnionitis	7 (3.6%)
Histological chorioamnionitis	7 (3.6%)
Antenatal steroids: none	13 (6.8%)
Antenatal steroids: complete courses only, mean (SD)	127 (66.4%)
Mode of birth: c-section	120 (62.8%)
**Maternal race**	
Asian	14 (7.3%)
Black	43 (22.5%)
North American Indian/Native Alaskan	4 (2.0%)
Other	12 (6.2%)
Unknown	44 (23.0%)
White	74 (38.7%)
Maternal Ethnicity: Hispanic	29 (15.1%)
Maternal age (years), mean (SD)	31 (6.0)
Baby’s health insurance: Medicaid/public	74 (38.7%)
Baby’s health insurance: private	115 (60.2%)
Maternal hypertension	43 (22.5%)
Maternal hypertension: pre-existing	21 (10.9%)
Maternal asthma during pregnancy	15 (7.8%)
Antepartum hemorrhage	9 (4.7%)
Medications to prolong pregnancy	101 (52.8%)
Maternal tobacco smoking	3 (1.5%)
Maternal opiates	2 (1.0%)
Apgar 1 min, mean (SD)	4 (2.2)
Apgar 5 min, mean (SD)	6 (2.0)
Resuscitation at birth: bag & mask	144 (75.3%)
Resuscitation at birth: intubation	107 (56.0%)
Resuscitation at birth: chest compression	5 (2.6%)
Resuscitation at birth: epinephrine	1 (0.5%)
Resuscitation at birth: surfactant	14 (7.3%)
Prophylactic indomethacin, ibuprofen, acetaminophen	75 (39.2%)
**Patent ductus arteriosus (PDA)**	82 (43%)
Medical treatment only (% of those with PDA)	41 (50%)
Interventional ligation (% of those with PDA)	13 (16%)
Hydrocortisone for hemodynamic support	27 (14%)
Dexamethasone for BPD treatment	24 (13%)

## Data Availability

The datasets generated in the multi-center Pre-Vent study are available in the NHLBI’s Biologic Specimen and Data Repository Information Coordinating Center (BioLINCC biolincc.nhlbi.nih.gov). These data include demographic data, respiratory and other support, summary data on SpO_2_%, heart rate, apnea, periodic breathing, and outcomes at 36- and 40-weeks PMA. Data will remain available for 7 years after its release. From our single center study, including calculation of cardiorespiratory phase synchronization, tabulated results summarized in the manuscript will be released by the corresponding author on reasonable request.

## IMPACT:

- Cardiorespiratory phase synchronization (CRPS) trajectories, a marker of autonomic nervous system functio is provided for 175 extremely preterm infants across post-menstrual age (PMA) and chronological age (CA).
- CRPS initially declines but subsequently increases with advancing PMA and CA, irrespective of discharge status and respiratory and neurological outcomes at 40 weeks PMA.
- At all CAs after the initial decrease, CRPS was higher for infants born at 27–28 weeks compared to those born earlier.
